# In Silico Molecular Dynamics of Griseofulvin and Its Derivatives Revealed Potential Therapeutic Applications for COVID-19

**DOI:** 10.3390/ijms23136889

**Published:** 2022-06-21

**Authors:** Parisa Aris, Masoud Mohamadzadeh, Yulong Wei, Xuhua Xia

**Affiliations:** 1 Department of Biology, Faculty of Science, University of Ottawa, 30 Marie Curie, P.O. Box 450, Station A, Ottawa, ON K1N 6N5, Canada; paris045@uottawa.ca; 2 Department of Chemistry, Faculty of Sciences, University of Hormozgan, Bandar Abbas 71961, Iran; masoud.mohamadzadehh@gmail.com; 3 Department of Microbial Pathogenesis, Yale University School of Medicine, New Haven, CT 06519, USA; yulong.wei@yale.edu; 4 Ottawa Institute of Systems Biology, University of Ottawa, Ottawa, ON K1H 8M5, Canada

**Keywords:** COVID-19, griseofulvin, griseofulvin derivatives, docking, drug repurposing, molecular dynamics, SARS-CoV-2

## Abstract

Treatment options for Coronavirus Disease 2019 (COVID-19) remain limited, and the option of repurposing approved drugs with promising medicinal properties is of increasing interest in therapeutic approaches to COVID-19. Using computational approaches, we examined griseofulvin and its derivatives against four key anti-SARS-CoV-2 targets: main protease, RdRp, spike protein receptor-binding domain (RBD), and human host angiotensin-converting enzyme 2 (ACE2). Molecular docking analysis revealed that griseofulvin (CID 441140) has the highest docking score (–6.8 kcal/mol) with main protease of SARS-CoV-2. Moreover, griseofulvin derivative M9 (CID 144564153) proved the most potent inhibitor with −9.49 kcal/mol, followed by A3 (CID 46844082) with −8.44 kcal/mol against M protease and ACE2, respectively. Additionally, H bond analysis revealed that compound A3 formed the highest number of hydrogen bonds, indicating the strongest inhibitory efficacy against ACE2. Further, molecular dynamics (MD) simulation analysis revealed that griseofulvin and these derivatives are structurally stable. These findings suggest that griseofulvin and its derivatives may be considered when designing future therapeutic options for SARS-CoV-2 infection.

## 1. Introduction

The COVID-19 pandemic has had an overwhelming long-term impact on healthcare systems globally. Despite vaccine developments and clinical research progress, the fast rate at which SARS-CoV-2 and its variants have spread has become a serious concern worldwide [1,2]. The World Health Organization reported over 529 million confirmed cases and over six million deaths as of 5 June 2022. During the last week of April 2022, the number of new weekly cases in the Americas and Africa increased by 9% and 32%, respectively. Remarkably, the number of new weekly deaths in the South East Asia increased by 41%. Despite a declining global trend in new cases over the first week of June, the total number of COVID-19 cases and deaths remains high.

Although extensive efforts have been made to manage this crisis, treatment of COVID-19 remains an urgent need and therapeutic options remained limited [3]. So far, different enzymes necessary for viral replication, including 3C-like main protease (3CLpro), papain-like protease (PLpro), and RNA-dependent RNA polymerase (RdRp), have been reported as potential treatment targets for SARS-CoV-2 infection [4,5]. Moreover, the SARS-CoV-2 spike protein receptor-binding domain (RBD) and its receptor angiotensin converting enzyme 2 (ACE2) are proposed as critical therapeutic targets for SARS-CoV-2 entry and infection [6,7].

Considering the time and cost required to develop new drugs, computational drug repurposing is an effective strategy for developing new indications for approved drugs already available for clinical profiles [8,9]. Griseofulvin is an FDA-approved drug that has primarily been used to treat dermatophyte infections [10,11]. Interestingly, it has received renewed attention in recent years, mainly due to its potential to treat cancer [12], and suppress the replication of the hepatitis C virus [13]. Griseofulvin inhibits cell division by acting on mitotic spindle microtubules, and induces cell death in cancer cell lines [12]. Additionally, griseofulvin arrests the G2/M phase in human cells by interacting with microtubule polymerization to suppress hepatitis C virus replication [13]. Recently, griseofulvin genes in the *gsf* gene cluster have been investigated among fungal genomes to identify essential genes involved in griseofulvin production [14].

In addition to these inhibitory effects, griseofulvin may also have function-enhancing effects by binding to ACE2. ACE2 participates in the regulation of the renin–angiotensin–aldosterone system (RAAS) [15]. Angiotensin I (ANG I) is converted by ACE to angiotensin II (ANG II), which increases blood pressure and inflammation. To maintain homeostasis and prevent tissue damage, ACE2 facilitates the breakdown of ANG II, thereby reducing blood pressure and inflammation [15]. For people with low levels of ACE2, ANG II tends to accumulate, with the deleterious effects of vasoconstriction, hypertension, and inflammation. Such patients are typically treated with an ACE inhibitor so that ANG I is not converted to ANG II [16]. SARS-CoV-2 binding to the ACE2 receptor tends to inhibit ACE2 function, resulting in excessive ANG II with its deleterious effects [17]. Based on this, blocking the ACE2 receptor could reduce intracellular viral penetration in the early stage of infection, but enhancing its functions, including vasodilation, reduced inflammation, and tissue damage, is desirable, especially in the most severe stages of SARS-CoV-2 infection [18]. Griseofulvin is long known to have the effect of vasodilation and improved capillary blood flow [19,20]. However, the molecular mechanism of griseofulvin on blood pressure remains unknown. Given the current understanding of ACE2, we hypothesize that griseofulvin may bind to ACE2, limiting further binding of SARS-CoV-2 with the extracellular domain of ACE2, and enhancing its vasodilation and tissue protection function.

Molecular docking provides preliminary information about small molecule interactions with receptors [21]. MD simulations seem to be pivotal in drug development, as they allow for the study of the behavior of proteins and other biomolecules in atomic resolution [22]. In the current in silico study, we performed molecular docking and molecular dynamics simulation analysis to investigate the inhibitory effects of griseofulvin and its derivatives in blocking essential SARS-CoV-2 M protease, RdRp, and RDB proteins (involved in its replication and propagation) and ACE2 from humans (involved in host cell entry). Our computational approaches evaluated the drug-likeness of griseofulvin and its derivatives against SARS-CoV-2. Our findings revealed that griseofulvin and its derivatives have good binding potential with both SARS-CoV-2 main protease, and the human ACE2 receptor, suggesting that these compounds may have inhibitory effects on SARS-CoV-2 entry and viral replication.

## 2. Results and Discussion

### 2.1. Virtual Screening and Molecular Docking Studies

**Figure 1:**
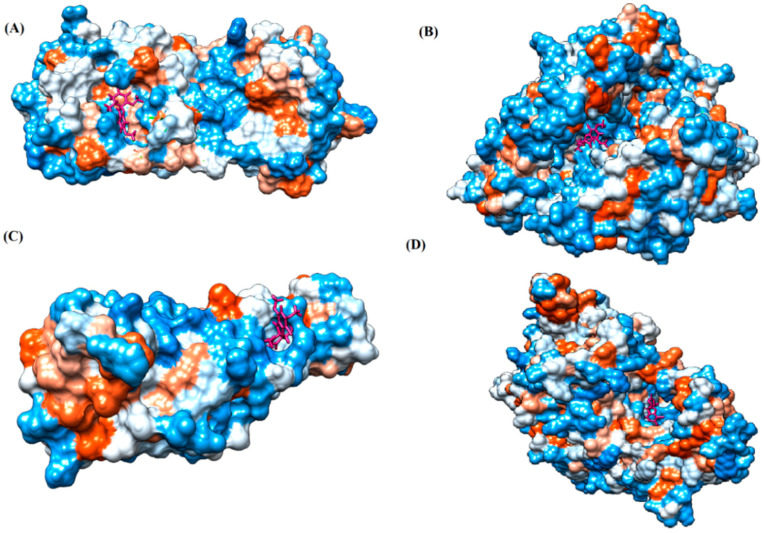
Ligand binding site prediction at (**A**) M protease, (**B**) ACE2, (**C**) RBD, and (**D**) RdRp hydrophobicity surface model, with ligand binding pockets in the protein structure. Dodger blue represents the most hydrophilic; the scale then moves to white, then to orange-red for the most hydrophobic regions. The ligand molecule (griseofulvin) is shown in bold, colored pink. Images were obtained by Chimera 1.15.

The proteins and ligand structures were optimized to be in their minimum energy state, which makes the molecule more stable. Molecules are most stable when their energy drops to a lower energy level [23]. Figure 1 depicts ligand binding site prediction of targeted proteins in the hydrophobicity surface model. In this study, Pyrx was used to score the docking quality of ligands and targeted SARS-CoV-2 proteins (Supplementary File S3). The structures were ordered by the lowest free energy values (kcal/mol), corresponding to the high affinity binding site. The top eight ligands were chosen for further analysis using AutoDock 4.2 (Supplementary File S4). The ligand with high affinity can change the conformation of the substrate and subsequently change its function. The structures with the highest binding affinities were mainly the compounds with the most significant interactions in hydrogen bonds. Of all interactions, most are shown between amino acids and ligands (Table 1), including hydrogen bonds (Green), alkyl and pi-alkyl (Pink), pi-cation (Orange), pi-sulfur (Yellow), Van der Waals interactions (Light Green), halogen (Blue), and carbon–hydrogen bonds (Gray).

#### 2.1.1. Main Protease

The coronavirus main protease (Mpro) is essential for the proteolytic maturation of the virus. It has been examined as a potential target to stop the spread of infection by inhibiting viral polyprotein cleavage [24,25]. The residues of His41, Gly143, Cys145, Glu166, Gln192, Asn142, Thr190, and Met165 6LU7 form H bonds with the carbonyl group, while Met49, Met165, Cys145, His41, and Pro168 show a π-alkyl interaction with pyridine. Phe140, Leu141, Asn142, Met165, Glu166, and Gln189 have van der Waals interactions, whereas Met165 and Cys44 show π-sulfur interactions with the SO2 group. Amino acids Gln189 and His41 form π-sigma and π-π-T shaped interactions, respectively. Moreover, there are halogen bonds between Arg188, Thr190, His164, His41, Glu166, and Gln181 with the chlorine of ligands. The amino acid interactions in the present study are similar to those in other studies that reported GLU166, CYS 44, CYS145, SER 144, and MET49 play roles in drug interactions [26].

Based on the lower dock score in the catalytic pocket of Main protease, the derivative M9 with -9.49 kcal/mol binding energy was the most effective, as it strongly binds to SARS-CoV-2 main protease, as confirmed by the AutoDock analysis. Notably, it has been reported that this derivative can be used to treat precancerous hyperproliferative conditions [27]. Our in silico study revealed that all structures showed good binding energy toward the target protein, ranging from −9.49 to −6.8 kcal mol^−1^.

#### 2.1.2. ACE2

The docking of receptor ACE2 with candidate ligands showed well established bonds with one or more residues in the active site of the receptor. The residues of Arg514, Glu402, Asn394, Ala348, Asp382, His401, Asp350, Gly394, and Phe400 form strong H-bond interaction with methylene and OH, while His378, Asp382, Asp350, Thr385, Ala348, Glu402, Glu398, and Asn394 are involved in weak van der Waals interactions. Close examination of the docking pose suggested a fluorine atom of A5 (PubChem ID 118254232), lined with the residues of Asp382 and Ala348. The molecular docking results also revealed that hydrophobic interactions of residues His401, Glu375, His378, Glu402, Glu398, His378, Ala 348, Arg514, and His345 are dominant interactions in the A1, A4, and A5 compounds, which have PubChem IDs 10574693, 118254131, 118254232, respectively.

The compound A3 (PubChem ID 46844082) exhibited the lowest binding energy, with the value of -8.44 kcal mol−1, indicating the highest affinity to the ACE2 receptor (Table 1). This compound is an analogue of griseofulvin, in which an n-propoxy group has replaced the methoxy group in the 2’ position; it exhibits a more substantial inhibiting effect on cancer cells than griseofulvin itself [28]. Two-dimensional receptor–ligand interaction diagrams illustrated that the A9 (PubChem ID 132286359) compound has the best interactions as hydrogenic (Asp382, Gly395, Phe400, His401, Glu402, Arg514) and hydrophobic (Glu375, His378, His401, Glu402) to ACE2 receptor. Since compounds A3 and A9 showed the highest binding affinity and interaction with ACE2, these complexes were considered for further MD simulation.

#### 2.1.3. Spike RBD

The amino acids Arg454, Arg457, Lys458, Ser459, Ile468, Se469, Glu471, and Ile472 can form hydrogen bonds in the binding cavity. Molecular docking results suggest that the hydrophobic actions of residues Arg454, Phe456, Arg457, Lys458, Arg466, Asp467, Ser469, Glu471, Ile472, Tyr473, and Pro491 with griseofulvin analogues played a predominant role in all compounds except Rb9, with the best result for the hydrogen bond interaction. The Rb3 compound showed the highest binding affinity towards the RBD protein, with a docking score of −7.21 kcal mol^−1^.

#### 2.1.4. RNA Dependent RNA Polymerase (RdRp)

The Rd2 compound showed a high binding affinity with −7.21 kcal mol^−1^ to the RdRp receptor (Table 1). The docking pose of Rd2 shows three hydrogen bonds interactions with the Lys621, Cys622, and Lys798 amino acids present at the active site of the RdRp protein. It is noteworthy that the H bonds in griseofulvin and the best interacting compound (PubChem ID 118254151) are identical to those found in Rd2 with the lowest binding energy. The selected ligand–protein complexes illustrated intermediate binding energy, ranging from 5.62–7.21 kcal mol^−1^ toward the target protein.

### 2.2. MD Simulations

**Figure 2:**
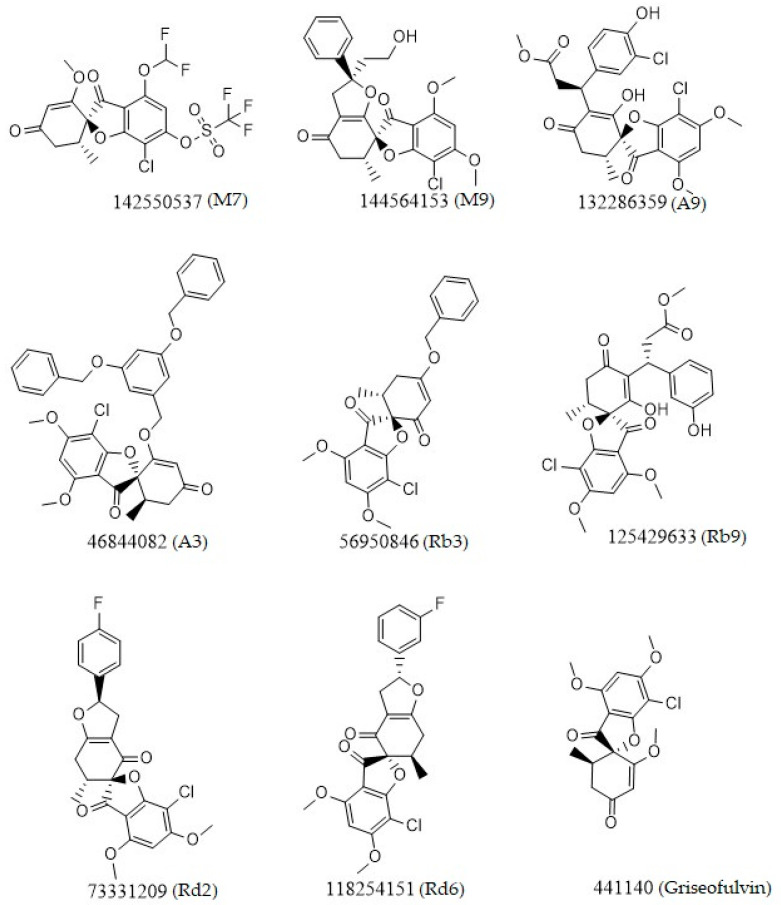
Chemical two-dimensional (2D) structures of the top ligands selected for MD analysis. The PubChem IDs are listed under the 2D structures.

The results in Table 1 suggest that the best inhibitors have strong hydrogen bonds with a large amount of non-covalent interaction. Figure 2 shows the two-dimensional (2D) structures of the best-docked ligand molecules, selected based on the lowest binding energy and highest number of hydrogen bonds. MD simulations were performed for 100 ns to analyze the protein structure stability and conformational changes of selected docked ligand complexes. The trajectory files from MD simulations were analyzed for root-mean-square deviation (RMSD), root mean square fluctuation (RMSF), radius of gyration (Rg), solvent-accessible surface area (SASA), secondary structure, and H bond occupancy, contributing to investigating the stability and structural dynamics during MD simulations. 

#### 2.2.1. Structural Dynamics of M protease

M protease is an essential component in the virus life cycle, as it coordinates viral replication and transcription. It cleaves polyproteins in their primary parts and releases replicative proteins. Consequently, this protein is considered a potential target for designing therapeutic drugs to counter SARS-CoV-2.

**Figure 3:**
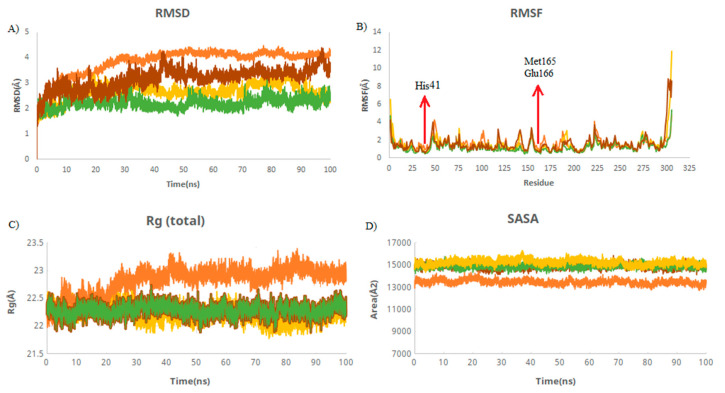
Structural dynamics of M protein. (**A**) Root mean square deviation (RMSD), (**B**) root mean square fluctuations (RMSF), (**C**) radius of gyration (Rg) plot, and (**D**) solvent-accessible surface area (SASA). Orange color indicates M protease-free form; yellow, green, and brown indicate M protease in a complex with M9, M7, and griseofulvin, respectively.

RMSD is used to measure the deviation between the initial and final positions of a protein. The smaller the RMSD, the more stable structures. The average RMSD values of M protease and M protease–griseofulvin were 4.12 and 3.51 Å, followed by the M7 (2.35 Å) and M9 (2.79 Å) compounds (Table 2). These values were found to be lower and remained stable during the simulations. This suggests that griseofulvin binds to M protease firmly and may block it. RMSF was used to depict the fluctuations at the residue level. The RMSF plot showed that residues of HIS41, Met165, and Glu166 interacting with the active site of M protease have the lower RMSF value compared to M protease alone (Figure 3B), indicating the stability of these residues upon binding to M protease during the simulation. The radius of gyration (Rg) indicates the compactness of the protein. The average Rg values of M protease and M protease–griseofulvin were revealed to be 22.9 and 21.8 Å, respectively (Table 2). This finding suggests that M protease–griseofulvin binds significantly more strongly than M protease alone. Protein-alone is a control used for comparison in this study. SASA is defined as the surface area of a protein which interacts with its solvent molecules. The average SASA values for M protease, M protease–griseofulvin, M7, and M9 were estimated to be 13,382, 14,834, 14,885, and 15,177 Å^2^, respectively. This plot suggests an increase in the total SASA of M protease, which means the internal residues in M protease are accessible to solvents when griseofulvin binds to them (Figure 3D).

#### 2.2.2. Structural Dynamics of Angiotensin-Converting Enzyme 2 (ACE2)

ACE2, a type I membrane protein, has a role in the renin–angiotensin–aldosterone system (RAAS), a key component in controlling hydroelectrolyte balance [29]. SARS-CoV-2 enters the host body via the respiratory tract and airway, where it binds to ACE2 receptors in human cells [30]. ACE2 is expressed in many human organs, including the lungs, heart, kidneys, bladder, and small intestine, and it demonstrates viral tissue tropism, which may lead to multiple organ failures [31,32,33].

**Figure 4:**
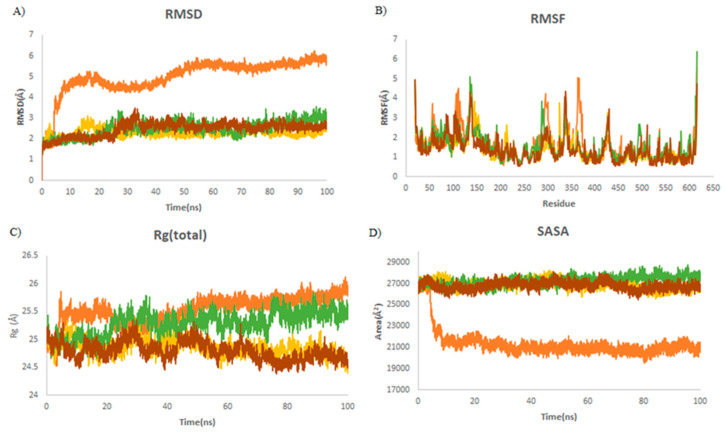
Structural dynamics of ACE2. (**A**) Root mean square deviation (RMSD), (**B**) root mean square fluctuations (RMSF), (**C**) radius of gyration (Rg) plot, and (**D**) solvent-accessible surface area (SASA). Orange color indicates ACE2-free form; yellow, green, and brown indicate ACE2 in a complex with griseofulvin, A9, and A3, respectively.

The RMSD trajectory measures protein deviation from the reference structure [34]. The binding of ligands into the receptor-binding pocket provides the conformational stability of the macromolecular system. In all simulations, the complex reaches an equilibrium during the last 20 ns of simulation, as is shown in Table 3. It was revealed that the average RMSD values were decreased by 3.4 Å (from 5.7 to 2.28 Å) upon binding to griseofulvin (Figure 4A). This finding shows the stability of griseofulvin in the active pocket of ACE2. There was minor increase in residual fluctuations. The average Rg values for ACE2 and the ACE2–griseofulvin, A3, and A9 compounds were found to be 25.7, 24.9, 24.7, and 25.5 Å. It was found that ACE2–A3 has more tight packing than ACE2 alone. The highest SASA value was observed in A9 (27,798 Å), followed by A3 (26,697 Å) and griseofulvin (26,532 Å), revealing that protein volume expansion and low variation is expected throughout the simulation time (Table 3).

#### 2.2.3. Structural Dynamics of RNA-Dependent RNA Polymerase (RdRp)

RdRp plays an essential role in the replication and transcription cycle of RNA viruses, making this protein an appealing antiviral drug candidate for a broad range of viruses [35]. Targeted inhibition of RdRp is unlikely to cause adverse effects due to its absence in mammalian cells [36,37,38]. Thus, repurposing of the RdRp-targeted drugs may represent a promising strategy for COVID-19 treatment [39,40]. 

**Figure 5:**
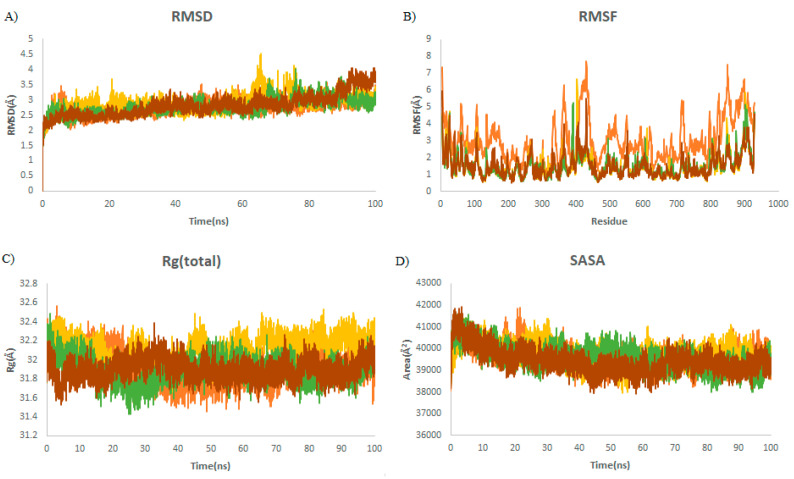
Structural dynamics of RdRp. (**A**) Root mean square deviation (RMSD), (**B**) root mean square fluctuations (RMSF), (**C**) radius of gyration (Rg) plot, and (**D**) solvent-accessible surface area (SASA). Orange color indicates RBD-free form; yellow, green, and brown indicate RdRp in a complex with griseofulvin, Rd6, and Rd2, respectively.

Steady RMSDs with small changes for almost all complexes demonstrated the stability of the complexes throughout the simulation (Figure 5A). No significant fluctuations in the average values of RMSFs were observed, which demonstrates the consistent complex exposure for a favorable conformational cavity of the RdRp molecule (Table 4). The average Rg values were found to be 32, 32.2, 31.9, and 31.9 Å for RdRp alone, RdRp–griseofulvin, Rd2, and Rd6, respectively (Figure 5C), which means that they have a similar structural packing to RdRp alone. The average SASA values of Rd6 (39,078 Å^2^), Rd2 (39,257 Å^2^), and griseofulvin (39,735 Å^2^) show no significant changes upon binding to RdRp (39,625 Å^2^). This indicates the slow rate of conformational changes in the protein, based on its solvent accessibility.

#### 2.2.4. Structural Dynamics of the Receptor-Binding Domain (RBD)

The receptor-binding domain in SARS-CoV-2 S protein binds to the human ACE2 receptor, enabling viral entry into the host cell [41,42]. The RBD is an attractive antigen for vaccine development, since it is the primary target for the majority of neutralizing antibodies produced during a SARS-CoV-2 infection [43].

**Figure 6:**
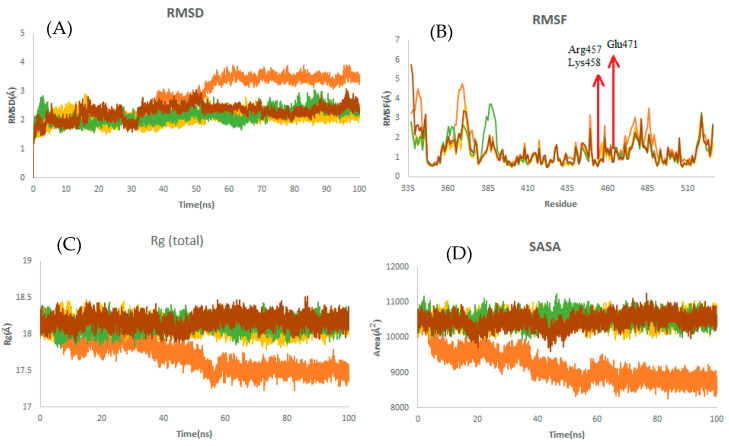
Structural dynamics of RBD. (**A**) Root mean square deviation (RMSD), (**B**) root mean square fluctuations (RMSF), (**C**) radius of gyration (Rg) plot, and (**D**) solvent-accessible surface area (SASA). Orange color indicates RBD-free form; yellow, green, and brown indicate RBD in a complex with griseofulvin, Rb9, and Rb3, respectively.

It is observed from Table 5 that the RBD–griseofulvin complex exhibits a lower RMSD (2.17 Å) than RBD alone (3.47 Å), implying the structural stability of griseofulvin in the active pocket of RBD. The average RMSF values of complexes during the last 20 ns of MD simulations indicate that the fluctuations in the constituent residues were less than 0.2 Å, which is satisfactory (Table 5). Furthermore, the residues Glu471, Lys458, and Arg457, which were found to interact with the binding site of RBD (Figure 6B), showed decreased RMSF values, indicating the most stable residues of this protein during the simulation. The average Rg values for RBD, RBD–griseofulvin, Rb9, and Rb3 were found to be 17.5 Å, 18.1 Å, 18.1 Å, and 18.2 Å, respectively (Figure 6C). The Rg plot shows that there is no significant difference in the packing of RBD upon ligand binding. From the SASA plot in Figure 6D, we can see that the SASA values of RBD–griseofulvin, Rb9 and Rb3 are overall greater than the RBD protein alone, leading to an increase in the solvent-accessible surface area, which results in the increased stability of the protein.

#### 2.2.5. Secondary Structure Changes upon Ligand Binding

The purpose of secondary structure is to identify protein structural characteristics. The average number of secondary structures in the complexes remained at approximately equal frequency over the last 20 ns of MD simulations, compared to protein alone, (Supplementary File S5). This finding suggests the stability of the secondary structure of the proteins upon binding to ligand.

#### 2.2.6. Hydrogen Bonding

Hydrogen bonds play an important role in the stability of the inhibitor–protein complex. To validate structural stability, the hydrogen bonds paired within 3.5 Å indicating the cutoff distance, as per the hydrogen bond, were calculated in a solvent environment during the 100 ns MD simulations (Table 6). Notably, the residue occupancies corresponding to the participation of active site residues demonstrated that the residues of Gln192 and Glu166 were predominant contributors, with 77.2% and 76.8% occupancies to the formation of H bonds with M protease during simulation. This finding highlights the significance of these residues in the structural state of the 6LU7 protein. The highest number of hydrogen bonds (six) was found between compound A9 and ACE2 as a receptor, followed by compounds M7 and M1, with five and four hydrogen bonds with M protease (6LU7), respectively, during the last 20 ns of MD simulations (Supplementary File S6). The results of H bond analysis are consistent with the docking findings, but the number of hydrogen bonds in the two approaches appear to be slightly different, which could be due to ligand–protein dynamics and conformation changes during MD simulation.

### 2.3. Drug-Likeness Evaluation

The ligands with the greatest potential for inhibiting targeted receptors were analyzed for their drug likeliness through SwissADME [44]. A compound seems to be more permeable and quickly absorbed by the body if it meets the Lipinski criteria [45]. Remarkably, all of the studied compounds except derivative A3 were shown to have a molecular weight ranging from 352.7 to 551.3 Daltons, with predicted LogP within the range value of 2.59–3.85. They all had less than ten hydrogen bond acceptors and five hydrogen bond donors. These characteristics are in the acceptable range of Lipinski’s rule of five (see Methods). Using PkCSM server, which evaluates hepatotoxic qualities, our results showed that none of the compounds exhibited hepatotoxicity. The calculated LogP values, hepatotoxicity, and other features of the bound ligands are illustrated in Table 1.

## 3. Materials and Methods

### 3.1. Ligand Preparation

The ligands’ 3D SDF file formats were obtained from PubChem, and PubChem CIDs were also retrieved. The PubChem CID of the ligand is 441140 for griseofulvin, and the CIDs of 464 griseofulvin derivatives are listed in Supplementary File S1. Open Babel version 2.4.1 was used to generate a co-crystallized ligand (Ligand.pdbqt) file and their optimization.

### 3.2. Receptor Preparation

The 3D protein structures of the receptors were downloaded from the RCSB Protein Data Bank (PDB) as.pdb files. The PDB IDs of the M protease, RdRp, spike RBD, and ACE2 receptor were 6LU7, 6M71, 6M0J (chain E), and 6M0J (chain A), respectively. Chimera version 1.15 (developed by the Resource for Biocomputing, Visualization, and Informatics, University of California San Francisco, San Francisco, CA, USA) was used for protein structure optimization. The structures were subjected to energy minimization for 1000 steepest descent steps at a root means square gradient of 0.02 Å, with an Amber ff14SB force field [46]. Due to the missing residues of the viral structures of the RdRp protein (PDB 6M71) in comparison with the complete amino acid sequences, the protein with PDB id 7AAP was retrieved from the protein data bank and was used as a template to predict a homology model of the RdRp protein using the SWISS-MODEL web server [47]. The stereochemical quality of the protein structure was validated using PROCHECK by Ramachandran plot [48,49]. The homology modeling results showed 100% homology to the 7AAP protein. The 7AAP protein is comprised of non-structural proteins 7, 8, and 12 (Nsp7-Nsp8-Nsp12) SARS-CoV2 RNA-dependent RNA polymerase, in complex with primer RNA duplex, along with favipiravir-triphosphate [50]. The Ramachandran plot of the modelled protein showed that 92% of residues lie in favored regions, followed by 8% in additional allowed regions (Supplementary File S2), indicating a good quality model.

### 3.3. Binding Site Prediction

The Computed Atlas of Surface Topography of proteins (CASTp) server was used to determine the binding sites of targeted proteins. The grid box was created around the identified binding pocket to perform virtual screening studies.

### 3.4. Virtual Screening and In Silico Molecular Docking

PyRx 0.8 [51] from MGLTools (https://ccsb.scripps.edu/mgltools/) was used for the virtual screening, using default settings. Ligands with the highest docking score were considered as the most stable conformation of the ligands regarding the receptor. Finally, eight hits with the highest docking score (most often negative) were selected for further analysis. Water molecules were manually removed from the PDB file prior to docking, and polar hydrogens were added. Molecular docking was performed for griseofulvin and thirty-two compounds using the Lamarckian genetic algorithm, conducted in AutoDock 4 [52]. Out of these modes, the two binding modes with the highest docking score and interaction with the selected protein were saved as a.pdb file and were used for further analysis. The interactions between the receptors and the ligands were visualized using Discovery Studio Visualizer v.20 (which can be freely downloaded from https://discover.3ds.com/discovery-studio-visualizer-download).

### 3.5. Molecular Dynamic Simulations

To validate the docking results, molecular dynamics simulation (MD) of the complex was performed. The single protein and two best protein–ligand and protein–griseofulvin complexes were analyzed using the GROMACS-2021 software package to carry out 100 ns simulations [53] with an AMBER99SB force field. The force field parameters for the ligands were generated by the AnteChamber PYthon Parser interface (ACPYPE) [54]. The TIP3P water model was selected for solvating complexes, followed by the addition of sodium and chloride ions to neutralize. Periodic boundary (PBC) conditions were used. Energy minimization was conducted at 1000 kJ/mol/nm. The system was equilibrated in the NVT and NPT ensembles for 1 ns. To achieve the overall charge neutrality of the systems and an ionic strength of 0.15 M, sufficient amounts of Na^+^ and Cl^−^ counter-ions were added instead of solvent molecules. To reduce significant forces and relax the simulated systems, an energy reduction procedure was used in the initial step, followed by a relaxation process. The Nose–Hoover thermostat and the Berendsen barostat were employed to keep the temperature and pressure at 310 K and 1.0 bar, respectively [55]. The trajectories were generated every 2 fs and saved every 2ps. Post-MD analyses were performed; these included root mean square deviation (RMSD), root mean square fluctuations (RMSF), the radius of gyration (Rg), solvent-accessible surface area (SASA), secondary structure, and hydrogen bond occupancy.

### 3.6. Drug-Like Properties of the Ligands

The drug likeliness of a molecule is indicated by Lipinski’s rule of five parameters (molecular weight less than 500 Da, no more than 5 hydrogen bond donors, number of hydrogen bond acceptors should be less than 10, and LogP should not be greater than 5) [45]. The SwissADME server (www.swissadme.ch/index.php) was used to obtain Lipinski’s rule of five parameters. Hepatotoxicity was also evaluated using the pkCSM server (http://biosig.unimelb.edu.au/pkcsm/prediction).

## 4. Conclusions

Several antiviral agents and other conventional treatments are being investigated as treatments for COVID-19, but therapeutic options remain poor [56,57]. The FDA-approved drug griseofulvin has gained much attention due to its low toxicity [58] and wide applications in treating and suppressing dermatophyte infections, cancer, and the hepatitis C virus [10,11,12,13]. Drug-likeness analysis of our studied compounds shows no violation of Lipinski’s rule of five, and no hepatotoxicity. Consequently, repurposing and developing derivatives of this compound might be a useful strategy for future therapeutic intervention.

Our in silico study results of the tested compounds showed good-to-significant activity toward target proteins of SARS-CoV-2. Griseofulvin exhibited the highest docking score, with the most interactions in the form of hydrogen bonds to COVID-19 main protease (6LU7), which is essential for the replication and transcription of the novel coronavirus (SARS-CoV-2). However, favorable interactions with ACE2, RBD, and RdRb were also identified for griseofulvin.

The molecular docking analysis revealed that, among the derivatives, M9, A3, Rb3, and Rd2, with docking scores greater than -7 kcal/mol, could act as the best inhibitors of SARS-CoV-2 main protease, ACE2, RBD, and RdRp, respectively. The structure of top ligands suggests that functional groups containing oxygen, such as the ether group in compounds M9, A3, and Rb3, may provide more possibilities for better interaction and higher affinity toward receptors. Moreover, structures with sulfonyl group in derivative M7, and fluorobenzene group in Rd2, could enhance the interaction between protein and ligand (Figure 2). The docked results were also supported by MD simulation analysis to affirm the stability of the studied complexes on the basis of RMSD, RMSF, Rg, hydrogen bonds (H-bond), and SASA values. These results outline the potential activity of griseofulvin and its derivatives as inhibitors of COVID-19. Nevertheless, further in vitro and in vivo investigations are required to validate the effectiveness of the proposed targets against SARS-CoV-2.

## Figures and Tables

**Table 1:** Molecular docking details of the top compounds selected for MD analysis. PubChem ID compound (first column), highlighted in red, blue, orange, and green, to indicate their interactions with M protease, ACE2, RdRp, and RBD, respectively. The dotted lines in the ligand interaction diagram (last column) show the key interacting residues the of targeted protein and ligand structure. Moreover, the green, light green, orange, pink, blue, and red dotted lines show conventional hydrogen bonds, van der Waals, cation–anion, pi-alkyl, halogen, and unfavorable acceptor–acceptor interactions, respectively.

PubChem ID	Binding Energy (kcal/mol)	H-Bonds	Hydrophobic Interactions	Lipinski Analysis		Ligand Interaction Diagram
441140 (M1)	−6.80	HIS 41 GLY 143 CYS 145 GLU 166	MET 49 MET 165 ARG 188	Molecular weight Lipophilicity H bond donor H bond acceptor Violations Lipinski Hepatotoxicity	352.7 2.95 0 6 0 Yes No	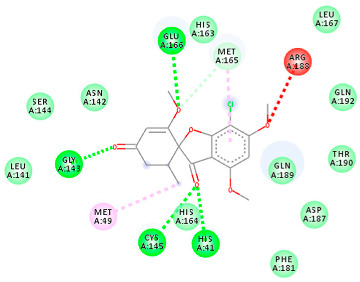
142550537 (M7)	−7.58	HIS 41 GLY 143 GLU 166 THR 190 GLN 192	MET 165 PRO 168 ARG 188 GLN 189 THR 190 GLN 192	Molecular weight Lipophilicity H bond donor H bond acceptor Violations Lipinski Hepatotoxicity	506.7 2.59 0 13 2 Yes No	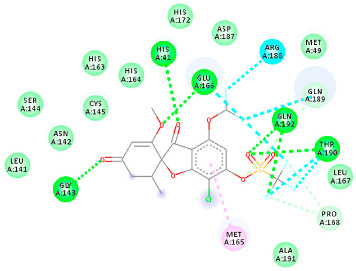
144564153 (M9)	−9.49	MET 165 GLU 166	CYS 44 MET 49 LEU 141 CYS 145 MET 165	Molecular weight Lipophilicity H bond donor H bond acceptor Violations Lipinski Hepatotoxicity	484.9 3.61 1 7 0 Yes No	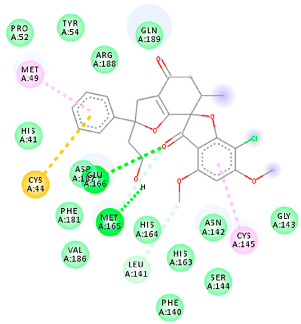
441140 (A7)	−5.2	HIS 401 ARG 514	HIS 378 GLU 398 HIS 401	Molecular weight Lipophilicity H bond donor H bond acceptor Violations Lipinski Hepatotoxicity	352.7 2.95 0 6 0 Yes No	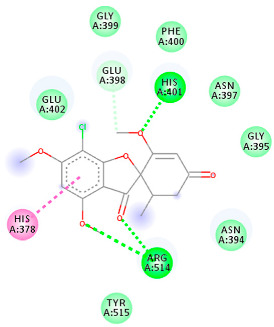
46844082 (A3)	−8.44	ALA 348 ASN 394 ARG 514	GLU 375 HIS 378 HIS 401 GLU 402	Molecular weight Lipophilicity H bond donor H bond acceptor Violations Lipinski Hepatotoxicity	641.1 5.51 0 8 2 Yes No	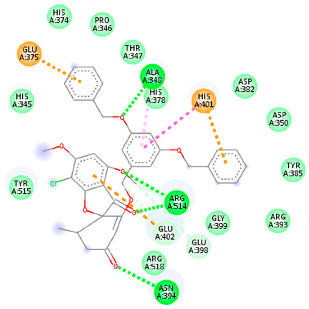
132286359 (A9)	−6.51	ASP 382 GLY 395 PHE 400 HIS 401 GLU 402 ARG 514	GLU 375 HIS 378 HIS 401 GLU 402	Molecular weight Lipophilicity H bond donor H bond acceptor Violations Lipinski Hepatotoxicity	551.3 3.85 2 9 1 Yes No	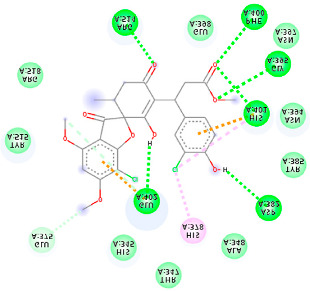
441140 (Rd7)	−5.62	LYS 621 CYS 622 LYS 798	ASP 623	Molecular weight Lipophilicity H bond donor H bond acceptor Violations Lipinski Hepatotoxicity	352.7 2.95 0 6 0 Yes No	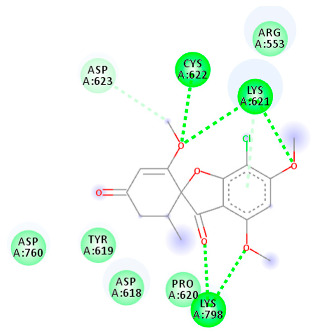
73331209 (Rd2)	−7.21	LYS 621 CYS 622 LYS 798	ASP 618 LYS 621 CYS 622 ASP 623	Molecular weight Lipophilicity H bond donor H bond acceptor Violations Lipinski Hepatotoxicity	458.8 3.75 0 7 0 Yes No	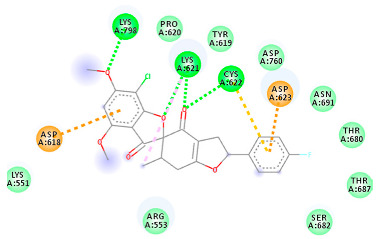
118254151 (Rd6)	−6.61	LYS 621 CYS 622 LYS 798	ASP 618 LYS 621 CYS 622 ASP 623 ASN 691	Molecular weight Lipophilicity H bond donor H bond acceptor Violations Lipinski Hepatotoxicity	458.8 3.73 0 7 0 Yes No	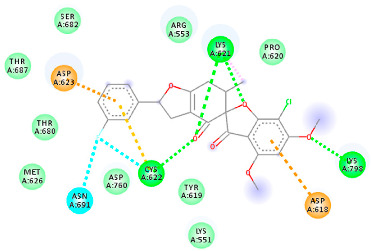
441140 (Rb1)	−5.3	GLU 471	LYS 458 GLU 471	Molecular weight Lipophilicity H bond donor H bond acceptor Violations Lipinski Hepatotoxicity	352.7 2.95 0 6 0 Yes No	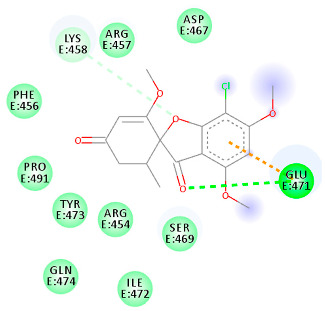
56950846 (Rb3)	−7.21	ARG 457 LYS 458 SER 469	ARG 457 LYS 458 GLU 471 PRO 491	Molecular weight Lipophilicity H bond donor H bond acceptor Violations Lipinski Hepatotoxicity	428.8 3.6 0 6 0 Yes No	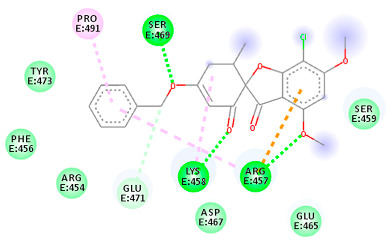
125429633 (Rb9)	−6.5	ARG 457 LYS 458 SER 459 GLU 471 ILE 472	ARG 457 LYS 458 GLU 471 PRO 491	Molecular weight Lipophilicity H bond donor H bond acceptor Violations Lipinski Hepatotoxicity	516.9 3.45 2 9 1 Yes No	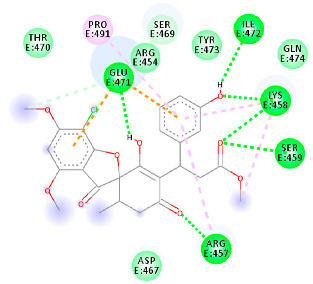

**Table 2:** The average of RMSD, RMSF, Rg, and SASA for M protease during the last 20 ns of MD simulations. PubChem IDs of derivatives M9 and M7 are 144564153 and 142550537, respectively.

Complex	Mean RMSD (Å)	Mean RMSF (Å)	Mean Rg (Å)	Mean SASA (Å)
M protease	4.12 ± 0.08	0.91 ± 0.40	22.99 ± 0.95	13,382 ± 199
M protease–M9	2.79 ± 0.30	1.2 ± 1.03	22.14 ± 0.11	15,177 ± 199
M protease–M7	2.35 ± 0.16	1.05 ± 0.49	22.30 ± 0.10	14,885 ± 180
M protease–griseofulvin	3.51 ± 0.23	1.17 ± 0.65	21.81 ± 0.11	14,834 ± 200

**Table 3:** The average of RMSD, RMSF, Rg, and SASA for ACE2 during the last 20 ns of MD simulations. PubChem IDs of derivatives A9 and A3 are 132286359 and 46844082, respectively.

Complex	Mean RMSD (Å)	Mean RMSF (Å)	Mean Rg (Å)	Mean SASA (Å)
ACE2	5.70 ± 0.15	0.99 ± 0.58	25.7 ± 0.09	20,933 ± 385
ACE2–A9	2.85 ± 0.21	1.18 ± 0.58	25.5 ± 0.11	27,798 ± 334
ACE2–A3	2.63 ± 0.13	1 ± 0.48	24.7 ± 0.9	26,697 ± 270
ACE2–griseofulvin	2.28 ± 0.12	1.12 ± 0.48	24.9 ± 0.11	26,532 ± 285

**Table 4:** The average of RMSD, RMSF, Rg, and SASA for RdRp during the last 20 ns of MD simulations. PubChem IDs of derivatives Rd6 and Rd2 are 118254151 and 73331209, respectively.

Complex	Mean RMSD (Å)	Mean RMSF (Å)	Mean Rg (Å)	Mean SASA (Å)
RdRp	2.9 ± 0.15	0.14 ± 0.06	32 ± 0.11	39,625 ± 380
RdRp–Rd6	3.1 ± 0.19	0.14 ± 0.06	31.8 ± 0.1	39,078 ± 389
RdRp–Rd2	3.34 ± 0.31	0.13 ± 0.05	31.9 ± 0.1	39,257 ± 318
RdRp–griseofulvin	3.1 ± 0.16	0.16 ± 0.07	32.2 ± 0.10	39,735 ± 381

**Table 5:** The average of RMSD, RMSF, Rg, and SASA for RBD during the last 20 ns of MD simulations. PubChem IDs of derivatives Rb9 and Rb3 are 125429633 and 56950846, respectively.

Complex	Mean RMSD (Å)	Mean RMSF (Å)	Mean Rg (Å)	Mean SASA (Å)
RBD	3.47 ± 0.12	0.95 ± 0.48	17.5 ± 0.06	8785 ± 128
RBD–Rb9	2.45 ± 0.18	1.2 ± 0.63	18.1 ± 0.07	10,514 ± 148
RBD–Rb3	2.41 ± 0.14	1.2 ± 0.63	18.2 ± 0.08	10,582 ± 152
RdRp–griseofulvin	2.17 ± 0.19	1.04 ± 0.50	18.1 ± 0.08	10,554 ± 155

**Table 6:** The residue interaction and hydrogen bond (H bond) percentage occupancy from the simulation trajectories.

Compound	Residue Interaction (H-Bond Occupancy (%))
6LU7-griseofulvin (M1)	Gln192 (17.2) Cys145 (43.4), Glu166 (6.1)
6LU7-142550537 (M7)	Gln192 (77.2), Thr190 (70.2), Gln189 (10.1)
6LU7-144564153 (M9)	Glu166 (76.8), His41 (3.8), His164 (15.1)
ACE2-griseofulvin (A7)	Trp69 (1.7), Ser106 (9.1), Asn117 (2.6), Lys114 (1.9)
ACE2-46844082 (A3)	Trp69 (2.2), Asn117 (24.2), Asn63 (1.15), Arg514 (10.2)
ACE2-132286359 (A9)	Trp69 (7.9), Asn51 (2.6), Asn53 (7.6), Gly104 (3.9)
RdRp-griseofulvin (Rd7)	Cys622 (37.2), Arg555 (17.2), Asp623(1.7)
RdRp-73331209 (Rd2)	Arg553 (2.3), Lys621 (2.1), Asp623 (9.1)
RdRp-118254151 (Rd6)	Arg553 (8.8), Lys621 (1.8), Asp623 (1.52)
RBD-griseofulvin (Rb1)	Asn360 (1.6), Cys391 (2.3), Phe486 (2.8), Ala522 (3.8)
RBD-56950846 (Rb3)	Thr430 (1.4), Lys458 (5.1), Ser459 (39.1), Ala522 (7.8)
RBD-125429633 (Rb9)	Asn460 (6.4), Arg466 (6.8), Asn354 (3.8), Ser349 (3.5)

## Data Availability

Not applicable.

## Supplementary Materials

The following are available online at https://www.mdpi.com/article/10.3390/ijms23136889/s1.
