# Supplementary material for: In Silico Molecular Dynamics of Griseofulvin and Its Derivatives Revealed Potential Therapeutic Applications for COVID-19
Source: Int J Mol Sci. 2022 Jun 21;23(13):6889. doi: 10.3390/ijms23136889 (PMC9267096; doi:10.3390/ijms23136889)
Supplement: Supplementary file 1 [file ijms-23-06889-s001.zip › File S2.pdf]

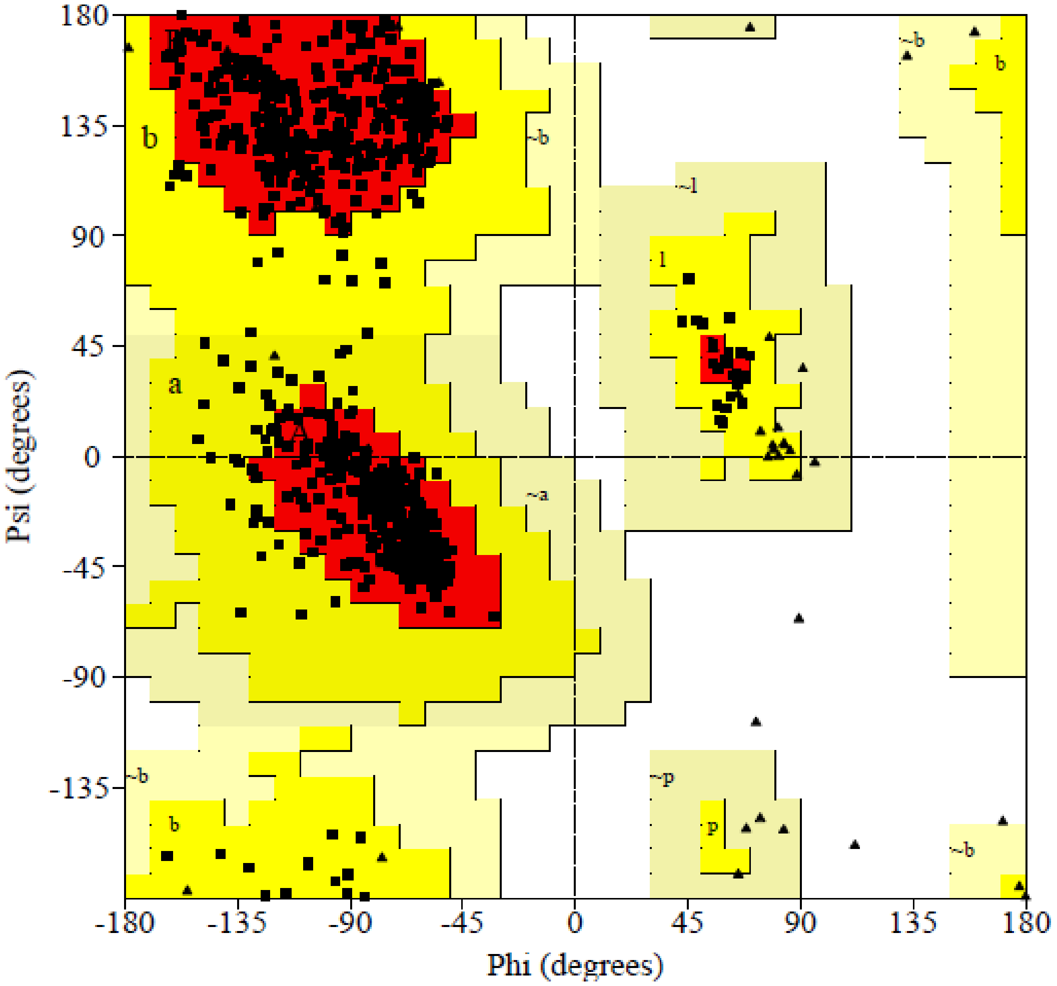

Figure S1. Ramachandran plot analysis of RdRp protein. Here, red region indicates favored region, yellow region for allowed and light yellow shows generously allowed region and white for disallowed region. Phi and Psi angels determine torsion angels.

Table S1. Ramachandran plot statistics of RdRp.

**Ramachandran plot statistics**	**RdRp**	
	**Residue**	**%**
Residues in the most favored regions [A,B,L]	960	92.00%
Residues in the additional allowed regions [a,b,l,p]	84	8%
Residues in the generously allowed regions [a,b,l,p]	0	0%
Residues in the disallowed regions [xx]	0	0%
Number of non-glycine and non-proline residues	1044	100%
Number of end residues (excl. Gly and Pro)	11	
Number of glycine residues (shown as triangles)	48	
Number of proline residues	35	
Total number of residues	1138	
