# Supplementary material for: In Silico Molecular Dynamics of Griseofulvin and Its Derivatives Revealed Potential Therapeutic Applications for COVID-19
Source: Int J Mol Sci. 2022 Jun 21;23(13):6889. doi: 10.3390/ijms23136889 (PMC9267096; doi:10.3390/ijms23136889)
Supplement: Supplementary file 1 [file ijms-23-06889-s001.zip › File S4, Table S2.pdf]

Table S2. Molecular docking details of ligands interaction with Mprotease (6LU7).

PubChem ID	Binding Energy (kcal/mol)	H-bonds	Hydrophobic interactions	Ligand interaction diagram	Lipinski Analysis	
CID_25171849 (M2)	-8.86	GLY 143	HIS 41<br>MET 49<br>LEU 141<br>MET 165<br>GLU 166<br>HIS 172<br>GLN 189	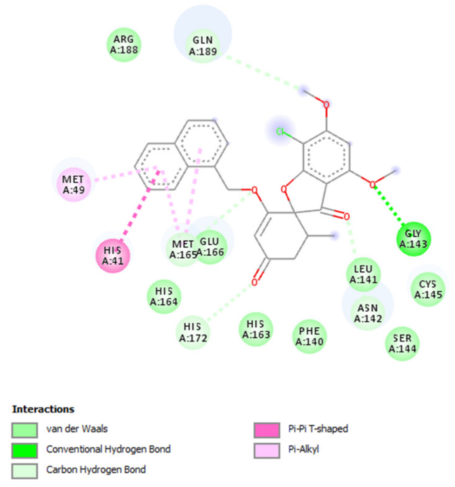	Molecular weight (<500 Da)<br>Lipophilicity (LogP<5)<br>H bond donor (<5)<br>H bond acceptor (<10)<br>Violations<br>Lipinski<br>Hepatotoxicity	478.9<br>3.29<br>0<br>6<br>0<br>Yes<br>No
CID_73330910 (M3)	-8.65	GLU 166<br>GLN 192	HIS 41<br>ASN 142<br>CYS 145<br>MET 165<br>PRO 168<br>GLN 189	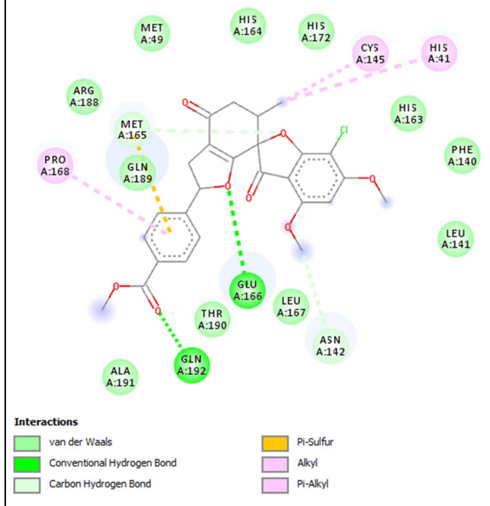	Molecular weight (<500 Da)<br>Lipophilicity (LogP<5)<br>H bond donor (<5)<br>H bond acceptor (<10)<br>Violations<br>Lipinski<br>Hepatotoxicity	498.9<br>4.08<br>0<br>8<br>0<br>Yes<br>No

Table S2. Molecular docking details of ligands interaction with Mprotease (6LU7).

CID_73331488 (M4)	-8.08	GLN 192	HIS 41<br>PHE 140<br>CYS 145<br>MET 165<br>GLU 166<br>PRO 168<br>GLN 189<br>THR 190<br>ALA 191	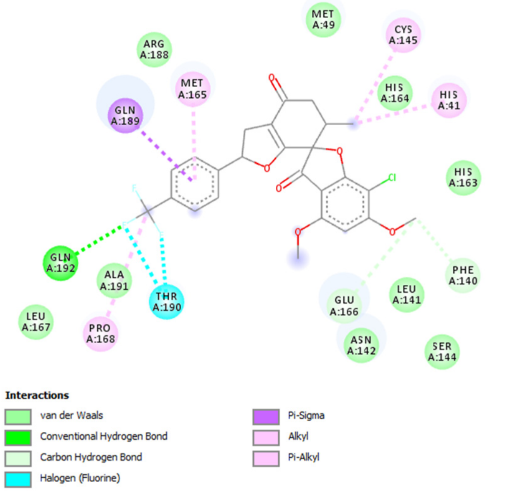	Molecular weight (<500 Da)<br>Lipophilicity (LogP<5)<br>H bond donor (<5)<br>H bond acceptor (<10)<br>Violations<br>Lipinski<br>Hepatotoxicity	508.8<br>4.00<br>0<br>9<br>1<br>Yes<br>No
CID_118254214 (M5)	-8.09	ASN 142<br>GLU 166	HIS 41<br>MET 49<br>PHE 140<br>LEU 141<br>HIS 164<br>MET 165<br>GLU 166	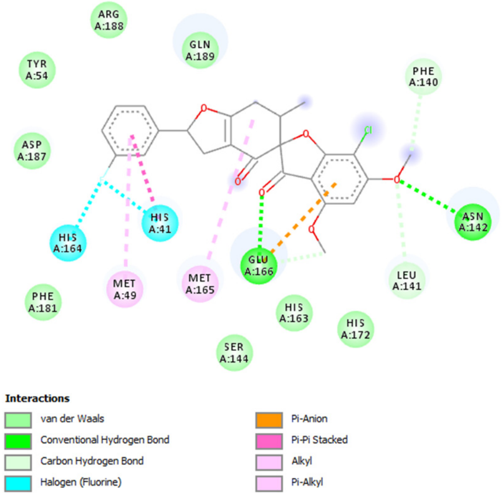	Molecular weight (<500 Da)<br>Lipophilicity (LogP<5)<br>H bond donor (<5)<br>H bond acceptor (<10)<br>Violations<br>Lipinski<br>Hepatotoxicity	458.8<br>3.83<br>0<br>7<br>0<br>Yes<br>No

Table S2. Molecular docking details of ligands interaction with Mprotease (6LU7).

CID_138490235<br>(M6)	-7.76	HIS 41<br>GLY 143<br>GLU 166<br>THR 190<br>GLN 192	MET 165<br>GLU 166<br>PRO 168<br>GLN 189<br>THR 190	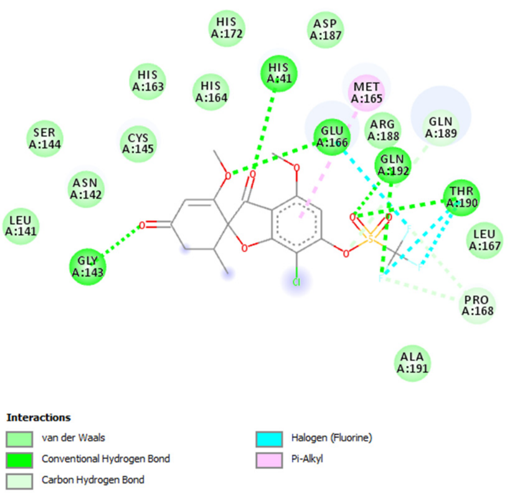	Molecular weight (<500 Da)<br>Lipophilicity (LogP<5)<br>H bond donor (<5)<br>H bond acceptor (<10)<br>Violations<br>Lipinski<br>Hepatotoxicity	470.8<br>2.84<br>0<br>11<br>1<br>Yes<br>No
CID_118254145<br>(M8)	-8.28	GLU 166	HIS 41<br>CYS 145<br>MET 165	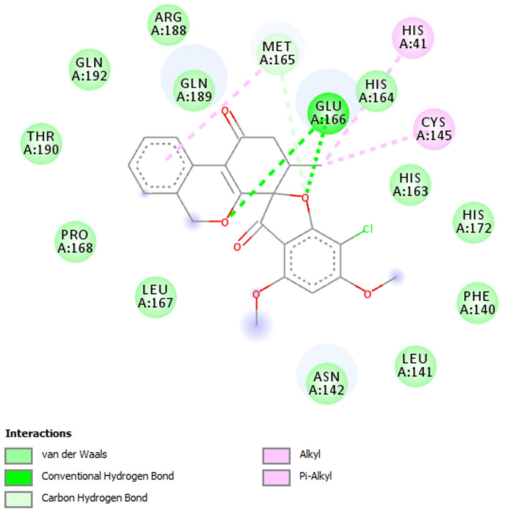	Molecular weight (<500 Da)<br>Lipophilicity (LogP<5)<br>H bond donor (<5)<br>H bond acceptor (<10)<br>Violations<br>Lipinski<br>Hepatotoxicity	426.85<br>3.45<br>0<br>6<br>0<br>Yes<br>No
