# Supplementary material for: In Silico Molecular Dynamics of Griseofulvin and Its Derivatives Revealed Potential Therapeutic Applications for COVID-19
Source: Int J Mol Sci. 2022 Jun 21;23(13):6889. doi: 10.3390/ijms23136889 (PMC9267096; doi:10.3390/ijms23136889)
Supplement: Supplementary file 1 [file ijms-23-06889-s001.zip › File S4, Table S3.pdf]

Table S3. Molecular docking details of ligands interaction with ACE2.

PubChem ID	Binding Energy (kcal/mol)	H- bonds	Hydrophobic interactions	Ligand interaction diagram	Lipinski Analysis	
CID_10574693 (A1)	-6.66	ARG 514	ALA 348<br>ASP 350<br>HIS 378<br>ASP 382<br>TYR 385<br>ASN 394<br>GLU 398<br>HIS 401	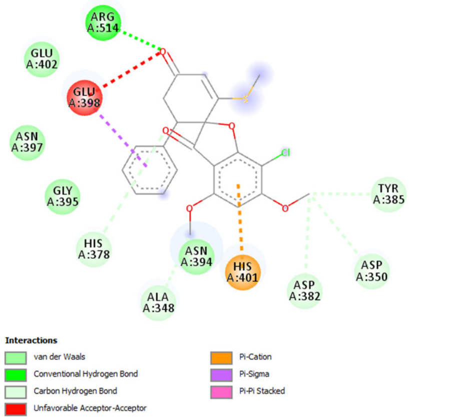	Molecular weight (<500 Da)<br>Lipophilicity (LogP<5)<br>H bond donor (<5)<br>H bond acceptor (<10)<br>Violations<br>Lipinski<br>Hepatotoxicity	430.9<br>3.39<br>0<br>5<br>0<br>Yes<br>No
CID_44566075 (A2)	-5.85	GLU 402<br>ARG 514	GLU 375<br>HIS 401<br>GLU 402	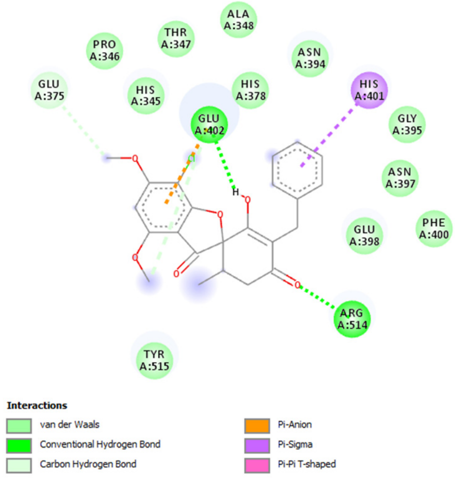	Molecular weight (<500 Da)<br>Lipophilicity (LogP<5)<br>H bond donor (<5)<br>H bond acceptor (<10)<br>Violations<br>Lipinski<br>Hepatotoxicity	428.8<br>3.25<br>1<br>6<br>0<br>Yes<br>No

Table S3. Molecular docking details of ligands interaction with ACE2.

CID_118254131 (A4)	-5.74	ARG 514	ALA 348<br>HIS 378<br>ASN 394<br>GLU 398<br>HIS 401<br>GLU 402<br>ARG 514	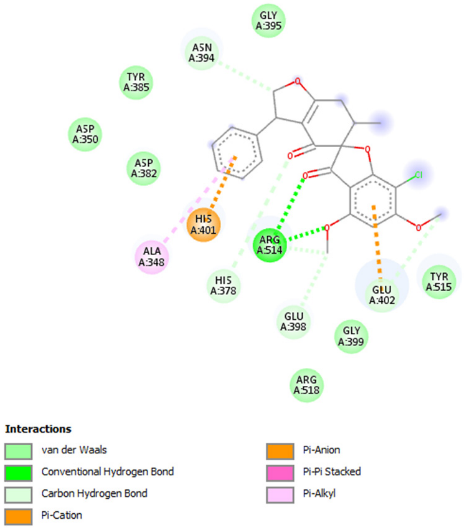	Molecular weight (<500 Da)<br>Lipophilicity (LogP<5)<br>H bond donor (<5)<br>H bond acceptor (<10)<br>Violations<br>Lipinski<br>Hepatotoxicity	440.8<br>3.58<br>0<br>6<br>0<br>Yes<br>No
CID_118254232 (A5)	-6.30	-	ALA 348<br>HIS 378<br>ASP 382<br>GLU 398<br>ARG 514	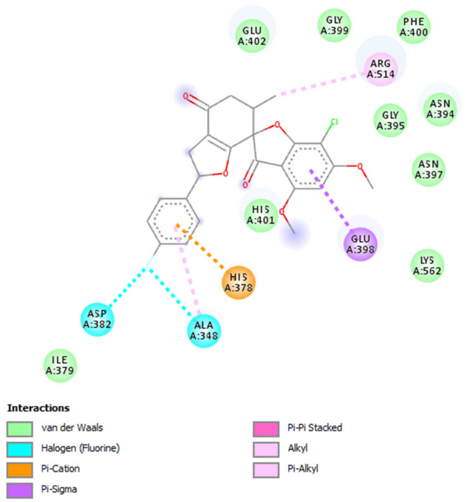	Molecular weight (<500 Da)<br>Lipophilicity (LogP<5)<br>H bond donor (<5)<br>H bond acceptor (<10)<br>Violations<br>Lipinski<br>Hepatotoxicity	458.8<br>3.76<br>0<br>7<br>0<br>Yes<br>No

Table S3. Molecular docking details of ligands interaction with ACE2.

CID_129895222 (A6)	-6.55	ALA 348<br>ASP 382	HIS 345<br>HIS 378<br>GLU 398<br>HIS 401	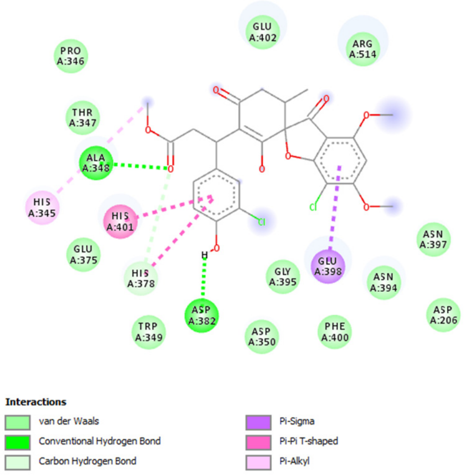	Molecular weight (<500 Da)<br>Lipophilicity (LogP<5)<br>H bond donor (<5)<br>H bond acceptor (<10)<br>Violations<br>Lipinski<br>Hepatotoxicity	551.37<br>3.61<br>2<br>9<br>1<br>Yes<br>No
CID_132286067 (A8)	-6.57	ALA 348<br>ASP 350<br>ASP 382<br>GLY 395	PRO 346<br>THR 347<br>HIS 378<br>HIS 401	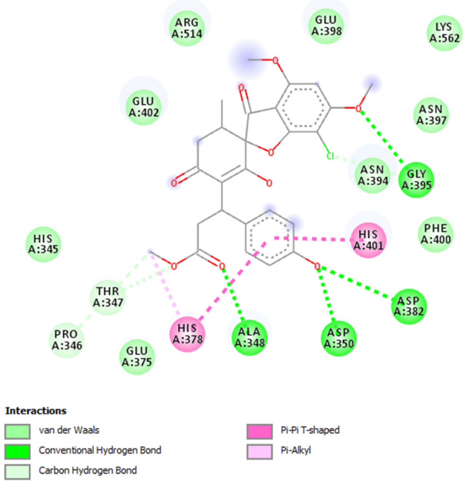	Molecular weight (<500 Da)<br>Lipophilicity (LogP<5)<br>H bond donor (<5)<br>H bond acceptor (<10)<br>Violations<br>Lipinski<br>Hepatotoxicity	516.9<br>3.52<br>2<br>9<br>1<br>Yes<br>No
