# Supplementary material for: In Silico Molecular Dynamics of Griseofulvin and Its Derivatives Revealed Potential Therapeutic Applications for COVID-19
Source: Int J Mol Sci. 2022 Jun 21;23(13):6889. doi: 10.3390/ijms23136889 (PMC9267096; doi:10.3390/ijms23136889)
Supplement: Supplementary file 1 [file ijms-23-06889-s001.zip › File S4, Table S4.pdf]

Table S4. Molecular docking details of ligands interaction with Rbd.

PubChem ID	Binding Energy (kcal/mol)	H-bonds	Hydrophobic interactions	Ligand interaction diagram	Lipinski Analysis	
CID_7331772<br>(Rb2)	-5.92	ARG 457<br>LYS 458<br>SER 459	ARG 454<br>ARG 457<br>LYS 458<br>ASP 467<br>GLU 471<br>PRO 491	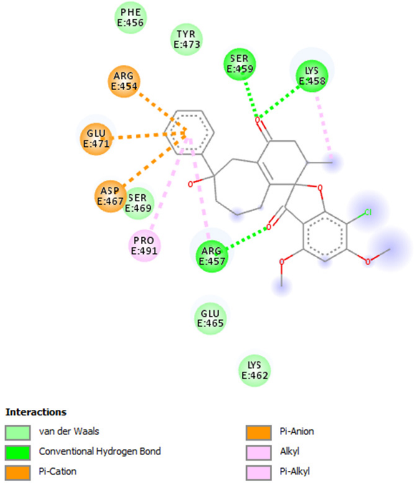	Molecular weight (<500 Da)<br>Lipophilicity (LogP<5)<br>H bond donor (<5)<br>H bond acceptor (<10)<br>Violations<br>Lipinski<br>Hepatotoxicity	417.5<br>3.47<br>0<br>5<br>0<br>Yes<br>No
CID_73330911<br>(Rb4)	-7.07	ARG 457<br>LYS 458<br>SER 459	ARG 454<br>ARG 457<br>SER 469<br>GLU 471<br>PRO 491	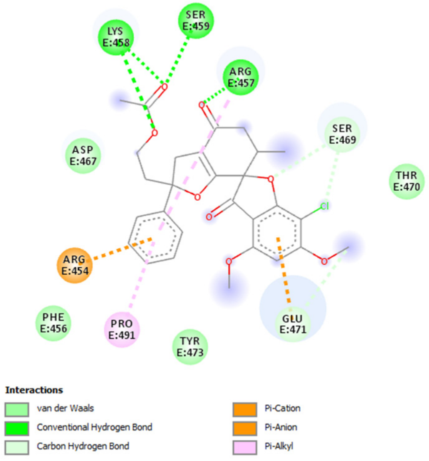	Molecular weight (<500 Da)<br>Lipophilicity (LogP<5)<br>H bond donor (<5)<br>H bond acceptor (<10)<br>Violations<br>Lipinski<br>Hepatotoxicity	526.9<br>4.27<br>0<br>8<br>1<br>Yes<br>No

Table S4. Molecular docking details of ligands interaction with Rbd.

CID_73331346 (Rb5)	-6.08	ARG 454<br>ARG 457<br>GLU 471	ARG 454<br>ARG 457<br>ASP 467<br>SER 469<br>GLU 471<br>PRO 491	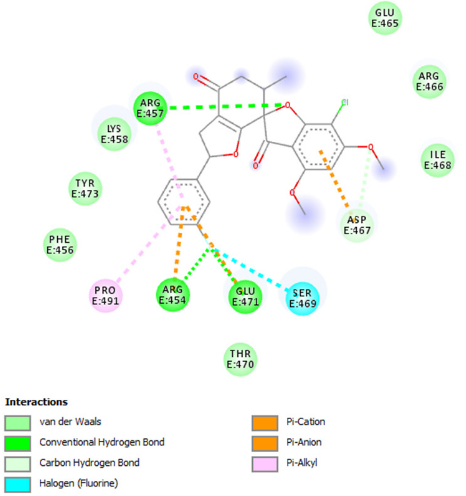	Molecular weight (<500 Da)<br>Lipophilicity (LogP<5)<br>H bond donor (<5)<br>H bond acceptor (<10)<br>Violations<br>Lipinski<br>Hepatotoxicity	458.8<br>3.73<br>0<br>7<br>0<br>Yes<br>No
CID_73331488 (Rb6)	-5.49	ILE 468<br>ARG 454	PHE 456<br>ARG 457<br>LYS 458<br>ASP 467<br>GLU 471<br>TYR 473<br>PRO 491	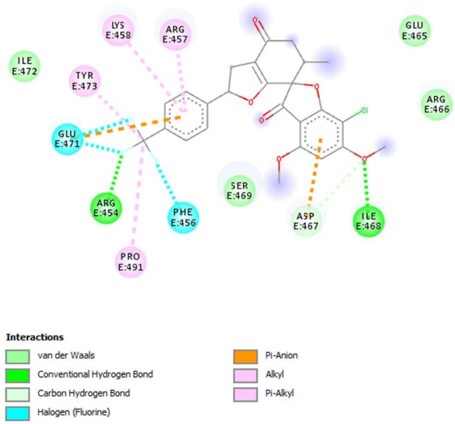	Molecular weight (<500 Da)<br>Lipophilicity (LogP<5)<br>H bond donor (<5)<br>H bond acceptor (<10)<br>Violations<br>Lipinski<br>Hepatotoxicity	508.8<br>4.00<br>0<br>9<br>1<br>Yes<br>No

Table S4. Molecular docking details of ligands interaction with Rbd.

CID_118254214 (Rb7)	-6.68	ARG 457<br>LYS 458<br>ILE 468<br>SER 469	ARG 466<br>ILE 472<br>TYR 473<br>PRO 491	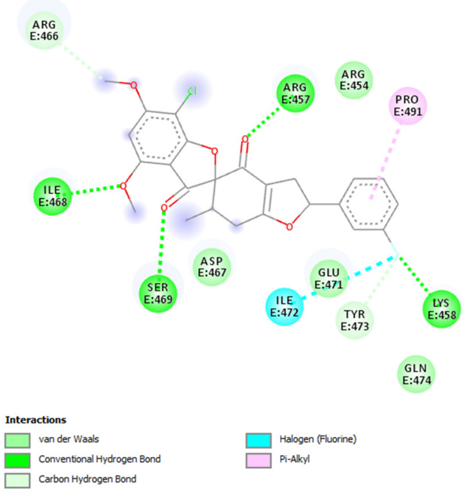	Molecular weight (<500 Da)<br>Lipophilicity (LogP<5)<br>H bond donor (<5)<br>H bond acceptor (<10)<br>Violations<br>Lipinski<br>Hepatotoxicity	458.8<br>3.83<br>0<br>7<br>0<br>Yes<br>No
CID_118263246 (Rb8)	-7.02	ARG 457<br>LYS 458<br>SER 459	ARG 454<br>ARG 457<br>ASP 467<br>SER 469<br>GLU 471<br>PRO 491	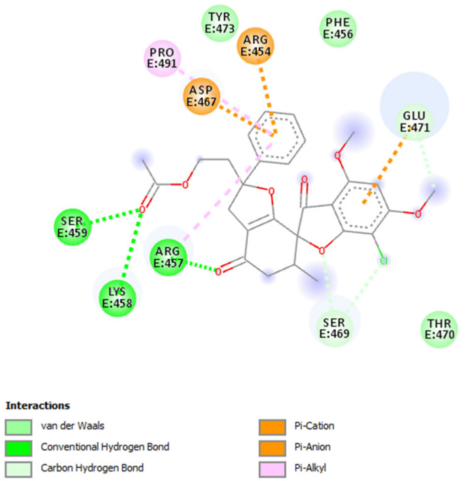	Molecular weight (<500 Da)<br>Lipophilicity (LogP<5)<br>H bond donor (<5)<br>H bond acceptor (<10)<br>Violations<br>Lipinski<br>Hepatotoxicity	526.9<br>4.27<br>0<br>8<br>1<br>Yes<br>No
