# Supplementary material for: In Silico Molecular Dynamics of Griseofulvin and Its Derivatives Revealed Potential Therapeutic Applications for COVID-19
Source: Int J Mol Sci. 2022 Jun 21;23(13):6889. doi: 10.3390/ijms23136889 (PMC9267096; doi:10.3390/ijms23136889)
Supplement: Supplementary file 1 [file ijms-23-06889-s001.zip › File S4, Table S5.pdf]

Table S5. Molecular docking details of ligands interaction with RdRp.

PubChem ID	Binding Energy (kcal/mol)	H- bonds	Hydrophobic interactions	Ligand interaction diagram	Lipinski Analysis	
CID_56924747<br>(Rd1)	-6.48	CYS 622	ASP 761<br>Glu 811<br>CYS 813	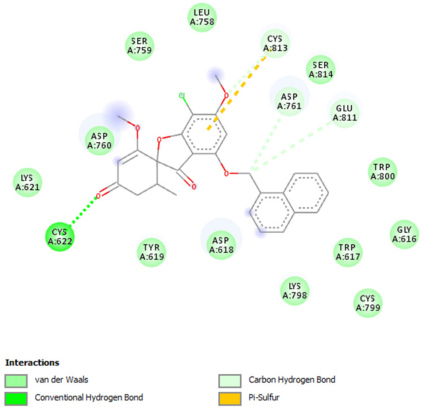	Molecular weight (<500 Da)<br>Lipophilicity (LogP<5)<br>H bond donor (<5)<br>H bond acceptor (<10)<br>Violations<br>Lipinski<br>Hepatotoxicity	478.9<br>3.68<br>0<br>6<br>0<br>Yes<br>No
CID_46844082<br>(Rd3)	-6.58	LYS 551<br>TYR 619<br>LYS 621	ASP 618<br>CYS 622<br>LYS 798	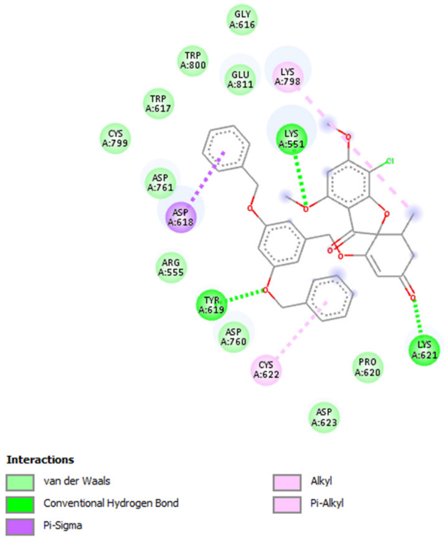	Molecular weight (<500 Da)<br>Lipophilicity (LogP<5)<br>H bond donor (<5)<br>H bond acceptor (<10)<br>Violations<br>Lipinski<br>Hepatotoxicity	641.1<br>5.51<br>0<br>8<br>2<br>Yes<br>No

Table S5. Molecular docking details of ligands interaction with RdRp.

CID_118254216<br>(Rd4)	-6.62	LYS 621<br>CYS 622<br>LYS 798	ASP 618<br>CYS 622<br>ASP 623	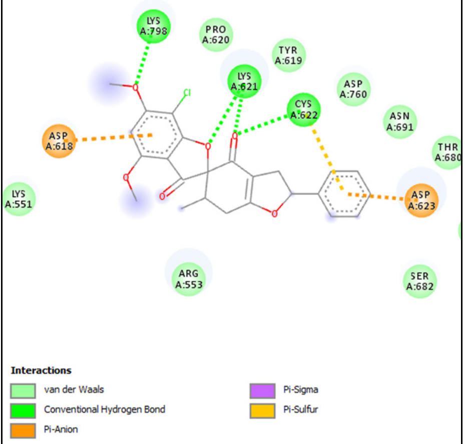	Molecular weight (<500 Da)<br>Lipophilicity (LogP<5)<br>H bond donor (<5)<br>H bond acceptor (<10)<br>Violations<br>Lipinski<br>Hepatotoxicity	440.8<br>3.65<br>0<br>6<br>0<br>Yes<br>No
CID_73331060<br>(Rd5)	-6.87	LYS 621<br>LYS 798	LYS 621<br>CYS 622<br>ASP 623	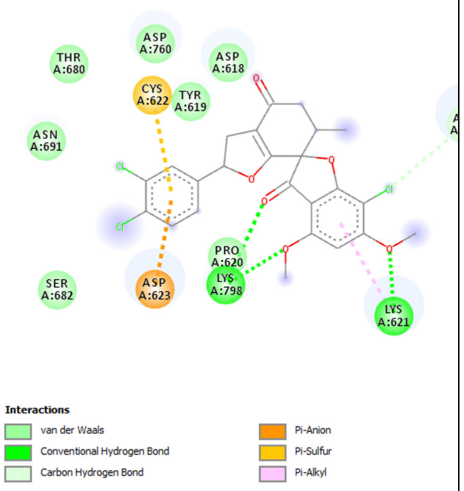	Molecular weight (<500 Da)<br>Lipophilicity (LogP<5)<br>H bond donor (<5)<br>H bond acceptor (<10)<br>Violations<br>Lipinski<br>Hepatotoxicity	509.7<br>4.09<br>0<br>6<br>1<br>Yes<br>No

Table S5. Molecular docking details of ligands interaction with RdRp.

CID_73331487<br>(Rd8)	-6.01	-	ASP 618<br>PRO 620<br>LYS 621<br>ASP 761	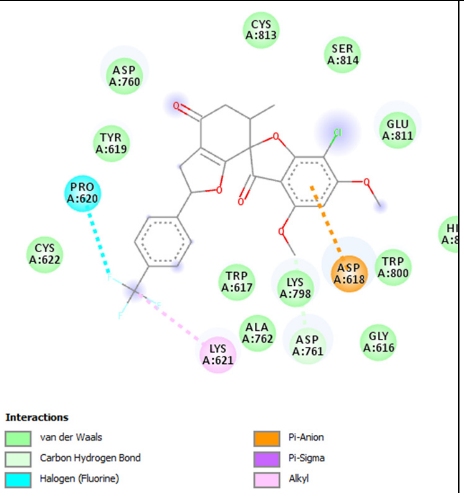	Molecular weight (<500 Da)<br>Lipophilicity (LogP<5)<br>H bond donor (<5)<br>H bond acceptor (<10)<br>Violations<br>Lipinski<br>Hepatotoxicity	508.8<br>4.01<br>0<br>9<br>1<br>Yes<br>No
CID_73330763<br>(Rd9)	-6.84	ARG 555	HIS 439<br>ILE 548<br>ALA 550<br>ASP 618<br>LYS 798	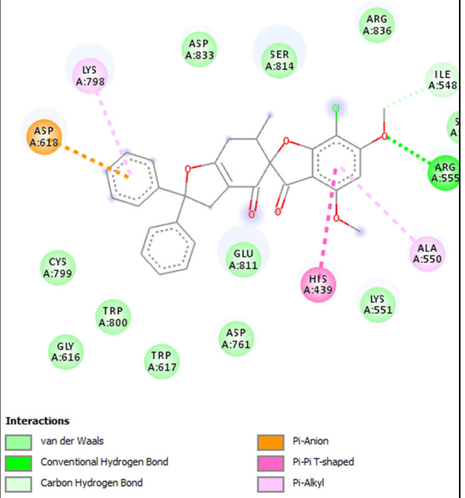	Molecular weight (<500 Da)<br>Lipophilicity (LogP<5)<br>H bond donor (<5)<br>H bond acceptor (<10)<br>Violations<br>Lipinski<br>Hepatotoxicity	516.9<br>4.22<br>0<br>6<br>1<br>Yes<br>No
