# Supplementary material for: In Silico Molecular Dynamics of Griseofulvin and Its Derivatives Revealed Potential Therapeutic Applications for COVID-19
Source: Int J Mol Sci. 2022 Jun 21;23(13):6889. doi: 10.3390/ijms23136889 (PMC9267096; doi:10.3390/ijms23136889)
Supplement: Supplementary file 1 [file ijms-23-06889-s001.zip › File S5.pdf]

Table S6. Secondary structure changes upon ligand binding.

Complex	Coil	B-Sheet	B-Bridge	Bend	Turn	A-Helix
6LU7	88.2±3.05	74.3±2.2	3.5±.95	32.8±3.4	47.6±4.4	53.2±3.2
6lu7-144564153	82.1±3.5	78.7±3.6	6.6±1.3	26.2±2.8	47.7±4.7	55.2±4.2
6lu7-142550537	81.2±2.4	80.9±2.1	6.1±0.6	22.06±3.2	52.5±4.9	51.7±5.1
6lu7-griseofulvin	79.1±2.9	79.2±2.4	5.2±0.98	33.6±3.1	45.2±4.9	51.5±4.3
ACE2	96.9±3.4	20.23±3.7	5.07±1.7	57.1±4.9	83.9±7.8	286.5±8.1
ACE2-132286359	97.6±3.2	23.35±2.7	3.89±1.1	56.1±4.6	68.8±7.2	326.7±6.4
ACE2-46844082	87.7±3.2	21.97±2.7	4.2±1.3	56.8±4.5	72.5±7.6	325.1±6.9
ACE2-griseofulvin	90.5±3.4	24.6±3.8	2.5±1.4	60.7±4.5	69.3±8.7	314±7.5
RBD	64.6±2.9	46.4±3.7	5.2±1.9	30.6±2.7	23.1±4.2	6.6±2.8
RBD-griseofulvin	59.6±2.3	52±2.3	3.8±1.4	26.2±2.3	23.9±4.2	8.41±3.25
RBD-125429633	64.8±2.8	47.3±2.6	4.5±1.5	28.4±2.9	22.8±3.75	8.75±3.5
RBD-56950846	61.4±2.4	51.8±2.4	3.1±1.5	28.2±2.5	22.3±4.1	9.2±3.6
RdRp	176.8±5	155.1±4.2	13.5±2.7	87.6±5.5	113.2±8.1	325±6.8
RdRp-griseofulvin	186.1±6	142.8±4.4	15.1±1.9	83.3±6.1	120.5±8.5	327.1±6.8
RdRp-118254151	185.6±6.6	139.5±6.3	14.4±2.5	89.1±5.5	105±7.6	335±6.3
RdRp-73331209	179.1±6.6	151.6±1.1	14.2±1.9	87.8±6.1	122.2±8.1	326.1±6.2
