# Supplementary material for: In Silico Molecular Dynamics of Griseofulvin and Its Derivatives Revealed Potential Therapeutic Applications for COVID-19
Source: Int J Mol Sci. 2022 Jun 21;23(13):6889. doi: 10.3390/ijms23136889 (PMC9267096; doi:10.3390/ijms23136889)
Supplement: Supplementary file 1 [file ijms-23-06889-s001.zip › File S6.pdf]

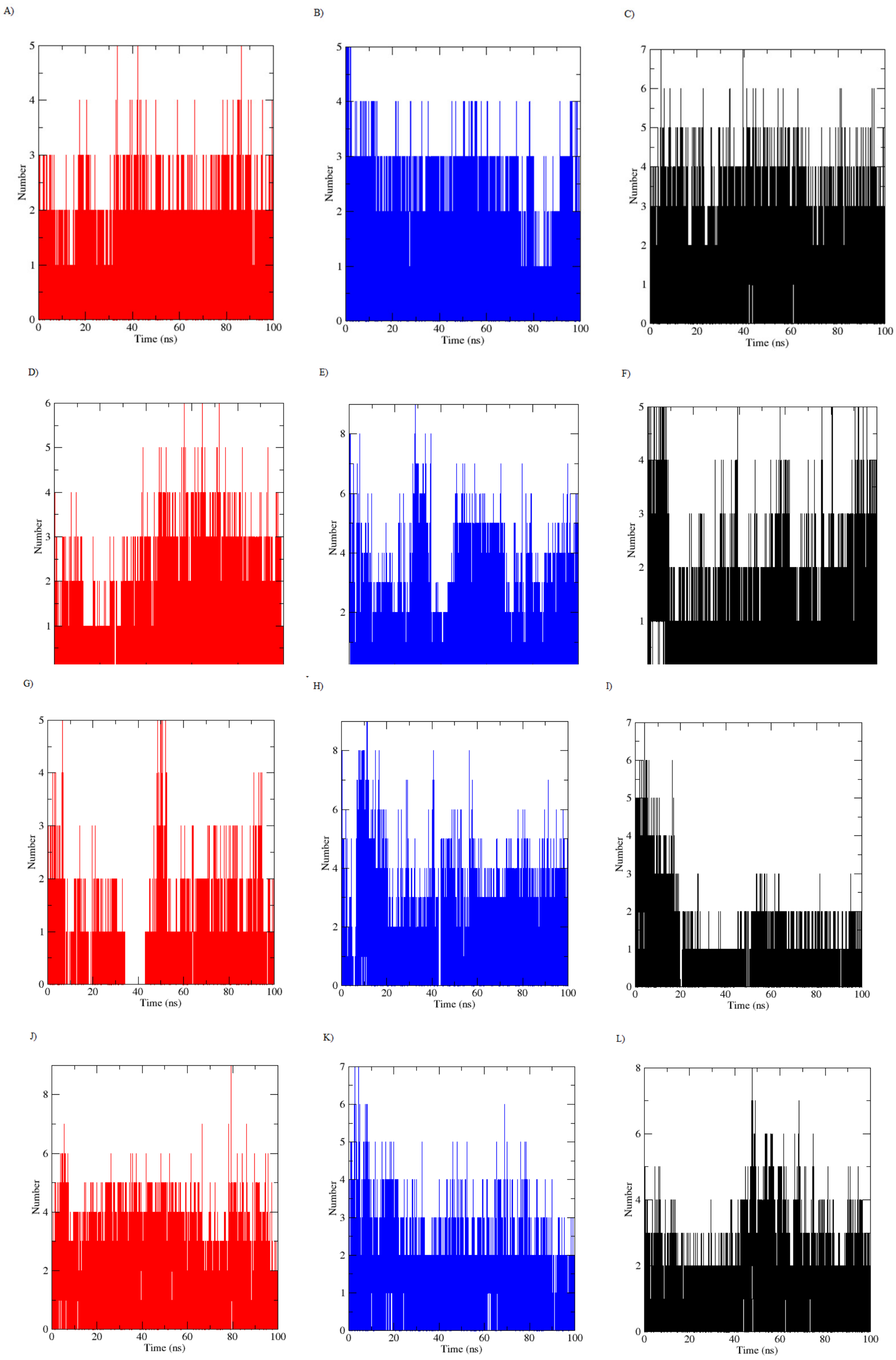

Figure S2. Hydrogen bonds analysis of best docked complexes. (A) Mprotease- griseofulvin (M1), (B) Mprotease-142550537 (M7), (C) Mprotease-144564153 (M9), (D) ACE2-griseofulvin (A7), (E) ACE2-132286359 (A9), (F) ACE2-46844082 (A3), G) RBD-griseofulvin (Rb1), (H) RBD-125429633 (Rb9), (I) RBD-56950846 (Rb3), (J) RdRp-griseofulvin (Rd7), (K) RdRp-73331209 (Rd2), (L) RdRp-118254151 (Rd6).
